# Where on the face do we look during phonemic restoration: An eye-tracking study

**DOI:** 10.3389/fpsyg.2023.1005186

**Published:** 2023-05-25

**Authors:** Alisa Baron, Vanessa Harwood, Daniel Kleinman, Luca Campanelli, Joseph Molski, Nicole Landi, Julia Irwin

**Affiliations:** ^1^Department of Communicative Disorders, University of Rhode Island, Kingston, RI, United States; ^2^Haskins Laboratories, New Haven, CT, United States; ^3^Department of Communicative Disorders, The University of Alabama, Tuscaloosa, AL, United States; ^4^Department of Psychological Sciences, University of Connecticut, Storrs, CT, United States; ^5^Department of Psychology, Southern Connecticut State University, New Haven, CT, United States

**Keywords:** audiovisual integration, phonemic restoration, speech perception, eye tracking, gaze patterns

## Abstract

Face to face communication typically involves audio and visual components to the speech signal. To examine the effect of task demands on gaze patterns in response to a speaking face, adults participated in two eye-tracking experiments with an audiovisual (articulatory information from the mouth was visible) and a pixelated condition (articulatory information was not visible). Further, task demands were manipulated by having listeners respond in a passive (no response) or an active (button press response) context. The active experiment required participants to discriminate between speech stimuli and was designed to mimic environmental situations which require one to use visual information to disambiguate the speaker’s message, simulating different listening conditions in real-world settings. Stimuli included a clear exemplar of the syllable /ba/ and a second exemplar in which the formant initial consonant was reduced creating an /*a*/−like consonant. Consistent with our hypothesis, results revealed that the greatest fixations to the mouth were present in the audiovisual active experiment and visual articulatory information led to a phonemic restoration effect for the /*a*/ speech token. In the pixelated condition, participants fixated on the eyes, and discrimination of the deviant token within the active experiment was significantly greater than the audiovisual condition. These results suggest that when required to disambiguate changes in speech, adults may look to the mouth for additional cues to support processing when it is available.

## 1. Introduction

In face-to-face interactions, the speaker’s message is typically seen (visible articulation on the face) and heard (the auditory signal). For many years, there has been evidence that visual information about speech influences what listeners hear, including increasing identification of the speech signal in the context of background noise (Sumby and Pollack, 1954; Grant et al., 1998). This influence of visual speech has been reported for a wide range of ages (Irwin et al., 2017a,b) for persons’ with typical and reduced hearing (Sommers et al., 2005), for clinical populations such as for persons with autism (Kuhl et al., 2005; Stevenson et al., 2014; Irwin et al., 2022), and for nonnative speakers of English (Reisberg et al., 1987). The presence of visual articulatory information can also facilitate the perception of heard speech, speeding up cortical processing of the auditory signal (van Wassenhove et al., 2005) and facilitating language processing (MacDonald et al., 2000; Lachs and Pisoni, 2004). Critically, the speaking face does not simply provide redundant information to the speech signal but can include functional cues that merge or influence the available auditory information to create a new percept. For example, the visible speech signal has been shown to change perception even when the auditory portion of the signal can be clearly heard, in the case of when mismatched auditory and visual speech results in a new “heard” percept (known as the McGurk effect; McGurk and MacDonald, 1976).

It is true that advantages to visual speech have been documented, however visual speech may not always prove useful to listeners. These situations may arise when listeners may be required to perceive subtle changes in speech sounds (Jordan and Thomas, 2011) or when listening to a nonnative language (Smiljanic et al., 2021). Additionally, audiovisual speech is not always available or used, such as instances of speaking on the telephone, situations in which the speaker is at a far distance, or even during face-to-face conversations for some listeners. For example, persons who are visually impaired exhibit speech production differences, suggesting that experience with a speaking face influences how speech is perceived as well as produced (Ménard et al., 2009). Furthermore, due to mask mandates during the COVID-19 global pandemic, the extent to which visible speech influences comprehension has become more apparent to listeners everywhere (Smiljanic et al., 2021). Masks create a barrier to articulatory information produced by the mouth (including lips, tongue, teeth, cheeks, and jaw). Masks also decrease loudness levels and can distort the clarity of the speech signal (Atcherson et al., 2020). Thus, it is important to investigate gaze patterns to a speaker when articulatory features are absent. By investigating gaze patterns within this context, we can better understand the factors that impact gaze to a speaker’s face to support speech understanding.

Previous studies which have investigated audiovisual perception in adults have done so in light of several communicative contexts. For example, in the context of speech in noise and in clear speech situations, studies have shown that adults mainly focus on the mouth of a speaker to support speech understanding (Vatikiotis-Bateson et al., 1998; Buchan et al., 2008). Yi A. et al. (2013) used eye tracking to measure eye gaze to the face in low and high signal-to-noise ratio (SNR) conditions. In low SNR conditions, listeners shifted their gaze to fixate more on the mouth of a speaker to support speech intelligibility. Hadley et al. (2019) measured eye gaze and movement patterns in an ecologically-friendly context in which conversational partners of varied hearing ability were required to hold conversations within quiet and noisy environments. Specifically, increased noise levels led to increased gaze to the speaker’s mouth, and conversational partners were more likely to move closer together to support understanding. Most recently, Banks et al. (2021) showed that adult listeners who were provided cues from a speaking face in the context of degraded speech signals demonstrated better recognition of speech than participants listening to degraded speech within an auditory only context. Banks et al. (2021) also found that longer fixations to the speaker’s mouth were related to better accuracy in the audiovisual condition. The previous studies indicate that as the task demands increased in the presence of background noise, gaze to the mouth was observed to increase as well. Research on audiovisual communication has extended into child populations with similar results. Irwin et al. (2017a,b) demonstrated that typically-developing children between the ages of 5–10 years show increased gaze to the mouth of a speaker in a range of audiovisual environments, including audiovisual speech, audiovisual speech in noise, and in an audiovisual mismatch condition.

Gaze patterns within both static contexts and movement contexts have also been examined. Much of the adult research suggests that when viewing a static face, gaze patterns typically form a “T” shaped pattern with most time allocated to the eyes, followed by the mouth or nose (Yarbus, 1967; Pelphrey et al., 2002). When viewing a speaking face, similar patterns emerge such that adults first fixate on the speaker’s eyes; however, once speaking begins, adult listeners then attend to the speaker’s mouth (Lansing and McConkie, 2003; Võ et al., 2012). It is important to know how gaze to a speaking face may change when environmental demands require the listener to actively discriminate between speech tokens compared to when passively viewing a speaking face. Active discrimination demands placed on the listener may be more generalizable to a real-time speaking situation in which a listener must process linguistic information to extract meaning from a spoken language.

Several studies have investigated audiovisual speech perception within the context of a full speaking face; however, fewer studies have investigated gaze patterns or speech perception in adults when articulatory features are unavailable or obscured (Jordan and Thomas, 2011; Irwin et al., 2017a). More recently, several studies have specifically investigated the impact that face masks have had on speech perception in different listening situations in adults (Brown et al., 2021; Smiljanic et al., 2021; Yi et al., 2021) and children (Lalonde et al., 2022) given the rise of mask wearing due to the COVID-19 pandemic. These studies collectively suggest that speech understanding is negatively impacted by masking, especially in the presence of noise, physical distancing, and nonnative speech. The use of transparent face masks can provide visual articulatory cues where listeners have a clear view of the speaker’s face and therefore support speech understanding, especially in the presence of noise (Yi et al., 2021; Lalonde et al., 2022).

The studies mentioned above have provided evidence that when visible articulatory information is absent, there can be a negative impact on speech perception. However, what is still unknown is the nature of patterns of gaze on a face when articulatory information is available and when it is not. Eye tracking can provide objective information on the nature of gaze patterns to a speaking face, to further our understanding regarding the specific types of visual information sought out by listeners to support speech understanding. Further, here we gain key information regarding the specific visual facial information sought out by listeners when visible articulatory information is absent. In previous studies, participants have been shown to optimize the processing of visual information by performing a series of fixations linked by saccades that shift foveal view to a new part of a visual scene (Yi A. et al., 2013). In experimental tasks, participants tend to fixate on areas in the visual scene that maximize the perceptual information needed to make optimal decisions (Barton et al., 2006; Peterson and Eckstein, 2013). Listeners, therefore, tend to look to articulatory movements of the mouth to support speech discrimination when the articulatory features are available. Eye tracking will allow for the measurement of specific gaze patterns when visible articulatory information is present (full speaking face is visible) or absent (articulatory information is unavailable). In this manner, eye tracking is leveraged to record how listeners gaze to different aspects of a speaking face across a range of listening conditions.

To investigate the influence of visual speech, our team has been using a specific paradigm based on the *phonemic restoration method* (Warren, 1970) to measure the influence of visible articulation on what is heard. Unlike speech in noise or McGurk-type stimuli, this method does not use auditory noise or overt category conflict, making it more like natural speech. In this method, two types of stimuli are presented to the listener: clear exemplars of an auditory consonant-vowel syllable (in this case, /ba/), and syllables in which the auditory cues for the consonant are substantially weakened, creating a stimulus which is more /a/−like, from this point on referred to as /*a*/. These stimuli are presented in an audiovisual context in which full features of the face, including the mouth and jaw are present to the viewer. In the audiovisual context, it is expected that the presence of the speaking mouth producing the bilabial consonant and vowel /ba/ will essentially restore the consonant, so that when participants hear /*a*/ they will perceive /ba/.^1^ In addition to the audiovisual condition, another condition with a pixelated mouth, was included in the experiments when presenting the stimuli /ba/ and /*a*/. The generalized movement in the area of the articulators can be perceived; however, true articulatory motion, such as the opening and closing of the mouth, is not available to the listener. Similar to wearing a mask, the area of the mouth was pixelated so that the articulation of the mouth was not visible. In the pixelated condition, phonetic restoration would not be expected.

This is important given the widespread use of masks during the COVID-19 pandemic, where listeners were in a communication situation when articulatory features were unavailable to the listener. Our paradigm is unique in that, to the author’s knowledge, no studies have directly investigated gaze patterns using eye-tracking technology to speaking faces where the mouth is present or absent, yet other facial features are present. Here we add to the literature by determining how listeners compensate for lost visual speech information by focusing on other facial features and the extent to which other facial features impact speech perception. Additionally, we investigate gaze patterns for these two conditions in the contexts of two experiments. The passive context requires simply observing the stimuli, whereas the active context requires the listener to press a button to identify the speech tokens. This allows us to investigate how gaze patterns are influenced by environmental demands for adults in different listening situations. Previous research has shown changes in gaze based on the inclusion or exclusion of noise, which can be considered a type of task demand (Yi A. et al., 2013; Hadley et al., 2019; Banks et al., 2021). Here, we extend these previous studies with a new type of task demand created by passive and active experiments. Given the different cognitive resources that may be needed to support effective communication, such as attention to relevant information, we investigated whether the listener’s gaze to the speaker’s face within audiovisual and pixelated condition varied depending on environmental demands, when the need to process the signal is increased due to an attenuated or distorted signal.

Within this paradigm we ask the following research questions:

1. How does the pattern of gaze differ to a speaking face differ in the audiovisual condition and pixelated condition?
2. How do the passive condition (in which the listener is only required to watch a speaking face) and active condition (which requires the participant to discriminate between speech tokens) affect the gaze pattern to a speaking face?
3. How do audiovisual and pixelated conditions impact accuracy levels of speech discrimination?

We hypothesize that within both the passive and active experiments, when audiovisual information from a speaking face is visible [audiovisual (AV) condition] participants will fixate mostly on the mouth (Vatikiotis-Bateson et al., 1998; Lansing and McConkie, 2003; Buchan et al., 2008; Võ et al., 2012; Yi L. et al., 2013; Yi A. et al., 2013; Hadley et al., 2019) however, they will do so more within the active experiment in which the participants are required to discriminate between speech tokens which maximizes the perception information needed for optimal decision-making (Banks et al., 2021). Within the passive and active experiments for the pixelated (PX) condition, we expect participants to gaze more at the eyes in both the passive and active experiments given that articulatory information from the mouth is unavailable and thus participants will attempt to glean any relevant perceptual information elsewhere (Yarbus, 1967; Pelphrey et al., 2002). In terms of accuracy data (only collected within the active experiment), we expect participants to demonstrate higher accuracy levels for the deviant contrast in the PX condition, where articulatory features from the mouth will not influence a phonemic restoration effect (Irwin et al., 2017a, 2022).

## 2. Materials and methods

### 2.1. Participants

Forty native English-speaking neurotypical adults (29 females, mean age = 24, SD = 7) were recruited in Connecticut and Rhode Island. To better characterize the sample of participants, information regarding race/ethnicity has been included. Of the participants 31 identified as White, 3 Black/African American, 3 Asian, 2 mixed race, 1 Hispanic, and 1 did not report. To be included in the study, all participants were monolingual English speakers with no known neurological impairment (e.g., history of concussions, seizures, epilepsy, traumatic brain injury, or neurodegenerative disorder). Audiological and visual screening were performed. Pure tone air conduction thresholds were screened in both right and left ears at frequencies from 500 to 4 K Hz using a portable Grason-Stadler GSI 18 screening audiometer and headphones at 25 dB. Participants were required to obtain a vision acuity screening using a multi-letter Snellen Eye Chart. Participants stood 20 feet from the chart which was posted on a wall and were required to read line 8 with both eyes open (aided with glasses if needed), which is the equivalent of 20/40 vision. All participants passed hearing and vision screenings.

A cognitive assessment was administered to characterize the sample and confirm typical perceptual reasoning skills. Cognitive testing was administered either the same day or in a separate session within a week of the experimental tasks so that all testing was completed within a close time frame. The *Weschler Abbreviated Scale of Intelligence-Second Edition* (WASI-2; Weschler, 2011), a brief measure of cognitive skill, was administered. Block Design and Matrix Reasoning subtests were administered which make up the Perceptual Reasoning composite score (M = 104.13, SD = 14.78, range = 83–148) and scores indicate neurotypical performance.

### 2.2. Procedure

Participants completed 1–2 sessions lasting approximately 1.5 h where they participated in both experimental procedures (eye tracking and EEG) and a behavioral assessment. This research was conducted as part of a larger study on phonemic restoration which included an EEG task. EEG and eye-tracking data were obtained simultaneously. As the research questions focus on the eye-tracking data, the EEG data will not be discussed here.

#### 2.2.1. Eye-tracking stimuli

The stimuli used in this study are identical to those in Irwin et al. (2017a,b). Stimuli were created by videotaping and recording an adult male speaker of English producing the syllable /ba/. The video was created in a sound attenuated room using a digital videocamera approximately 3 feet from the speaker. The auditory stimuli are created by synthesizing speech based on a natural production of the syllable and systematically flattening the formant transitions to create the /*a*/. Video of the speaker’s face does not change (always producing /ba/), but the auditory stimuli (/ba/ or /*a*/) vary. Thus, when the /*a*/ auditory stimulus is dubbed over the visual /ba/, a visual influence will effectively “restore” the weakened auditory cues so that the stimulus is perceived as a /ba/, akin to a visual phonemic restoration effect (Samuel, 1981; Kashino, 2006; Irwin et al., 2011, 2017a,b, 2022; Irwin and Brancazio, 2014).

**Figure 1:**
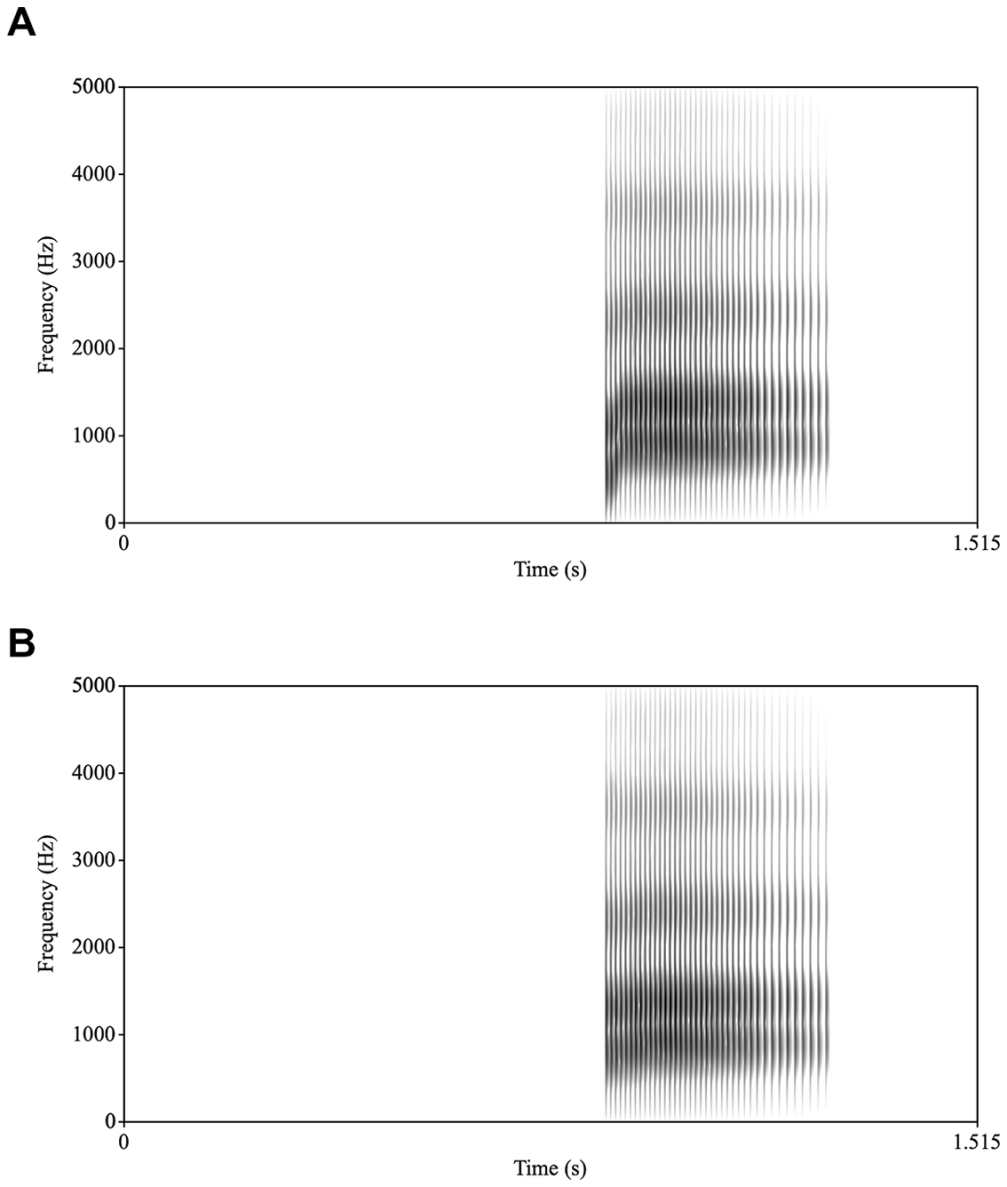
Spectrograms of **(A)** /ba/ and **(B)** /*a*/.

**Figure 2:**
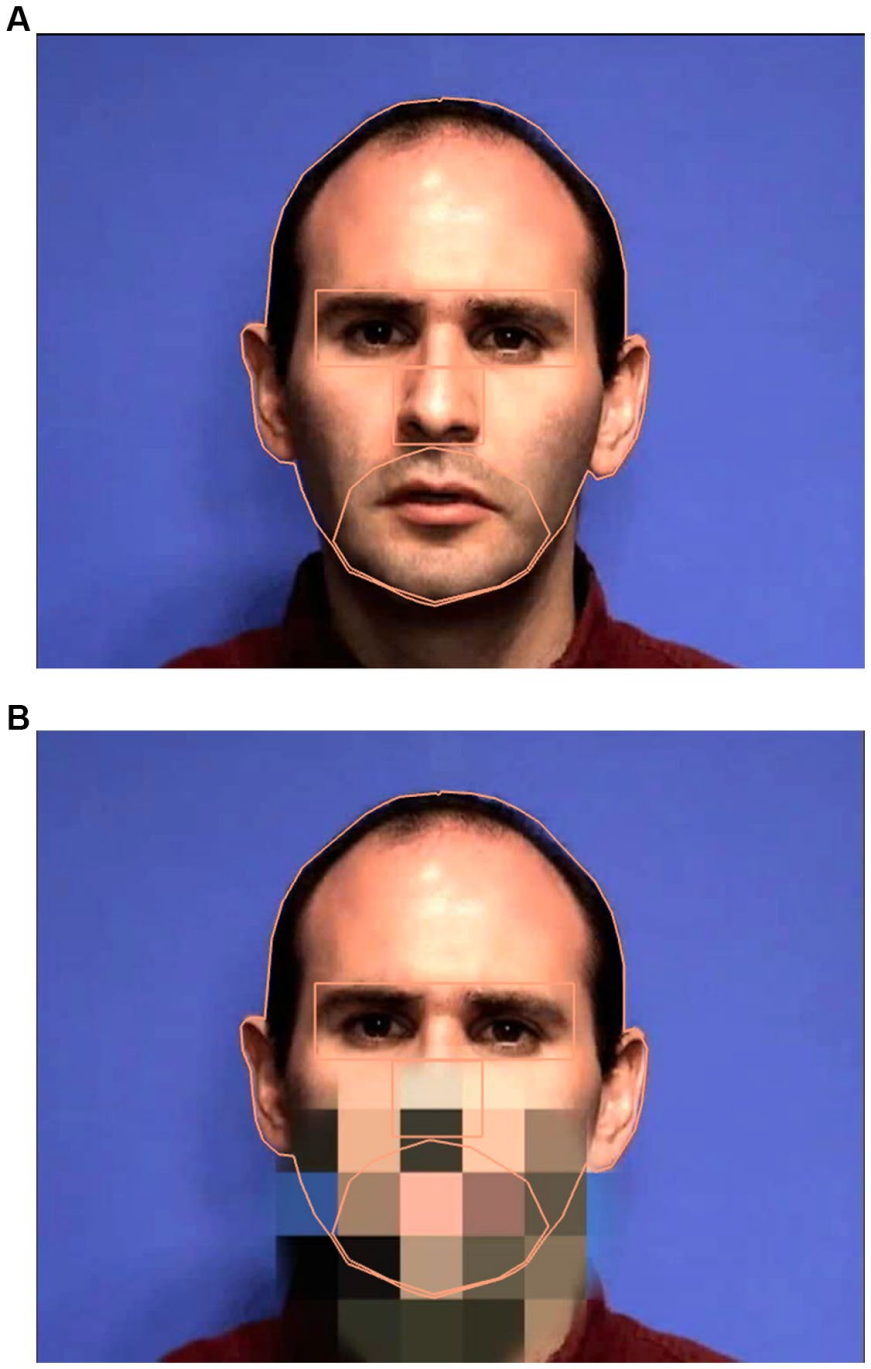
Sample images of the speaker when the mouth is visible **(A)** and when the mouth is pixelated **(B)** including the interest areas.

High quality audio was captured separately with Praat (version 6.2.14; Boersma and Weenink, 2016) and a Macbook Pro with a Sennheiser microphone. The microphone was placed centrally in front of the speaker approximately 2 feet away below the vediocamera. /ba/ tokens were repeated 5 times for 5 iterations for a total of 25 tokens. A single token was used as the basis for all stimuli. Using Praat, acoustic parameters were extracted for this token, including formant trajectories, amplitude contour, voicing, and pitch contour. The token had rising formant transitions for F1, F2, and to a lesser extent F3, characteristic of /ba/. To create the /ba/ stimulus, a new token of /ba/ was synthesized based on these values. To create the /*a*/ stimulus, the synthesis parameters were modified: the onset values were changed for F1 and F2 to reduce the extent of the transitions and lengthened the transition durations for F1, F2, and F3, and then a new stimulus was synthesized. For the /ba/, the transitions were 34 ms long and F1 rose from 500 Hz to 850 Hz; F2 rose from 1,150 Hz to 1,350 Hz; and F3 rose from 2,300 Hz to 2,400 Hz. For the /*a*/, the transitions were 70 ms long and F1 rose from 750 Hz to 850 Hz; F2 rose from 1,300 Hz to 1,350 Hz; and F3 rose from 2,300 Hz to 2,400 Hz (see Figure 1 for spectrograms of /ba/ and /*a*/). As the audio was recorded simultaneously but separately from the video, the /ba/ and /*a*/ synthesized auditory stimuli were dubbed (and aligned down to a single frame for accuracy) onto a video of the speaker producing /ba/ (audiovisual (AV) condition), with the acoustic onsets synchronized with the visible articulation or a video of a face with a pixelated mouth region with no visible movement (PX condition; see Figure 2). To create stimuli for the PX condition, the mouth portion of the video was reduced to 36 48 × 48 pixel solid blocks. The mouth region itself is contained within 9 of these blocks (3 × 3). This pixelation ensures that the articulatory movements of the mouth and jaw cannot be perceived (although variation in the pixelation indicates movement). All stimulus videos are publicly accessible at.^2^

#### 2.2.2. Eye-tracking procedure

The experiment was conducted in a windowless room. A five-point calibration and validation of the eye tracker was conducted for each participant prior to testing. Recalibration and validation were completed if there was a detected x- or y-axis drift over 2° (standard drift check when using Eyelink). Most participants did not have drift larger than 1.25°. Participants were instructed to look at the screen as looks off-screen would pause the experiment.

The experiment was created using Experiment Builder software (SR Research Experiment Builder 1.10.165). These stimuli were presented in an 70/30 oddball design in which /ba/ is the frequently occurring (or standard stimulus, 70% of the time), with /*a*/ serving as the infrequently occurring (or deviant stimulus, 30% of the time), in both face contexts. The first experiment was passive (no button press), and the stimuli were presented in AV and PX conditions. The second experiment was active (included a button press), and the stimuli again were presented in AV and PX conditions. Participants always completed the passive experiment first, followed by the active experiment. For both experiments, the AV condition was first (then the PX condition) to ensure that the phonemic restoration effect was tested without exposure to the contrast of the /ba/ and /*a*/ auditory tokens. The active experiment required participants to press a button on a gamepad to identify if they heard a standard or deviant stimulus. At the start of the active experiment, participants were played the deviant and standard sounds (/*a*/ and /ba/ respectively) and told which button on the gamepad (right or left) corresponded to each sound. This happened before each condition for the active experiments to remind participants what they were listening for as the buttons were not marked in order to avoid the potential for participants to look away from the monitor. Each condition (AV and PX) was 9 min and contained 200 trials (140 standard and 60 deviant) lasting 2000 ms each, for a total experiment time (4 total blocks/conditions) of 36 min. The interstimulus interval (ISI) between trials was 200 ms. The audio was adjusted to a comfortable listening-level which was approximately 65 dB. At the beginning of each block, a drift check was performed. Five practice trials were included at the beginning of each AV condition and at the beginning of each PX condition for both passive and active experiments.

### 2.3. Apparatus

Monocular data was collected across two sites – one at Haskins Laboratories with an Eyelink 1000 and one at the University of Rhode Island with an EyeLink Portable Duo with a sampling rate of 500 Hz for both systems. Stimulus sentences were presented on a 19-inch PC computer monitor in both sites with a display resolution of 1,280 × 1,024. Participants sat at a viewing distance between 80 and 90 cm. As EEG data and eye-tracking data were obtained simultaneously, the eye tracker remote mode was implemented and a target sticker was placed on the EEG cap on the participants’ forehead, allowing the system to compensate for head movements of up to 20 cm.

### 2.4. Data preprocessing

To preprocess the data, areas of interest were identified on the face. These include: eyes, nose, mouth/jaw, and the head. Using EyeLink Data Viewer (version 4.1.1), these areas were defined on the target face (Figure 2). The head interest area includes the entirety of the target head including the ears and hair. The eye interest area is a rectangle and defined from the superior portion of the brow to the top of the cheekbone, as in the literature, it is typical to combine results from both eyes in analyses (Pelphrey et al., 2002; Speer et al., 2007; Falck-Ytter, 2008; Jones et al., 2008). The mouth/jaw interest area was hand drawn, and superiorly meets the nose, including the philtrum, and inferiorly runs along the base of the jaw and runs laterally to the nasolabial sulcus. It is important to note that the mouth/jaw interest area was created to encompass the entire mouth, including the open posture/movement during test trials. The nose is defined by the inferior portion of the eye interest area and the superior region of the mouth/jaw interest area, laterally it extends the width of the nose.

### 2.5. Data cleaning procedure

The eye-tracking data was exported using EyeLink Data Viewer software and the data cleaning was conducted in several stages. One participant had over 100 trials with no onscreen fixations for the active condition. As this is over 25% of the collected data, this participant’s data was excluded from further analyses. The data was then cleaned using a 3-stage cleaning. Specifically, fixations of <80 ms and within 0.5° were merged with neighboring fixations, fixations of <40 ms and within 1.25° were merged with neighboring fixations, and any remaining fixations <80 ms were excluded from analysis. It has been found that 50 ms is the eye-to-brain lag and thus is considered the minimal duration a person must look at a stimulus to extract useful visual information for processing (Inhoff and Radach, 1998). Many researchers agree that short fixations reflect a microsaccade, a truncated fixation, a blink, or other artifact in the eye-movement data (Godfroid, 2020). Thus, it is best to merge or remove short fixations as they are not thought to reflect cognitive processing (Godfroid, 2020). After 3-stage cleaning, trials were removed if the participant did not have any onscreen fixations (0.8%) or no fixations on the face (2.0%).

### 2.6. Analysis

We sought to identify the patterns of gaze to a speaking face in two experimental contexts – one passive and one active. Within each experiment, there were two conditions where participants saw a full speaking face (audiovisual, AV condition) or a speaking face with a pixelated mouth (PX condition). Within the passive and active experiments and for both AV and PX conditions, two speech stimuli were presented, including a standard auditory /ba/ and an attenuated /*a*/ within both the AV and PX conditions.

**Figure 3:**
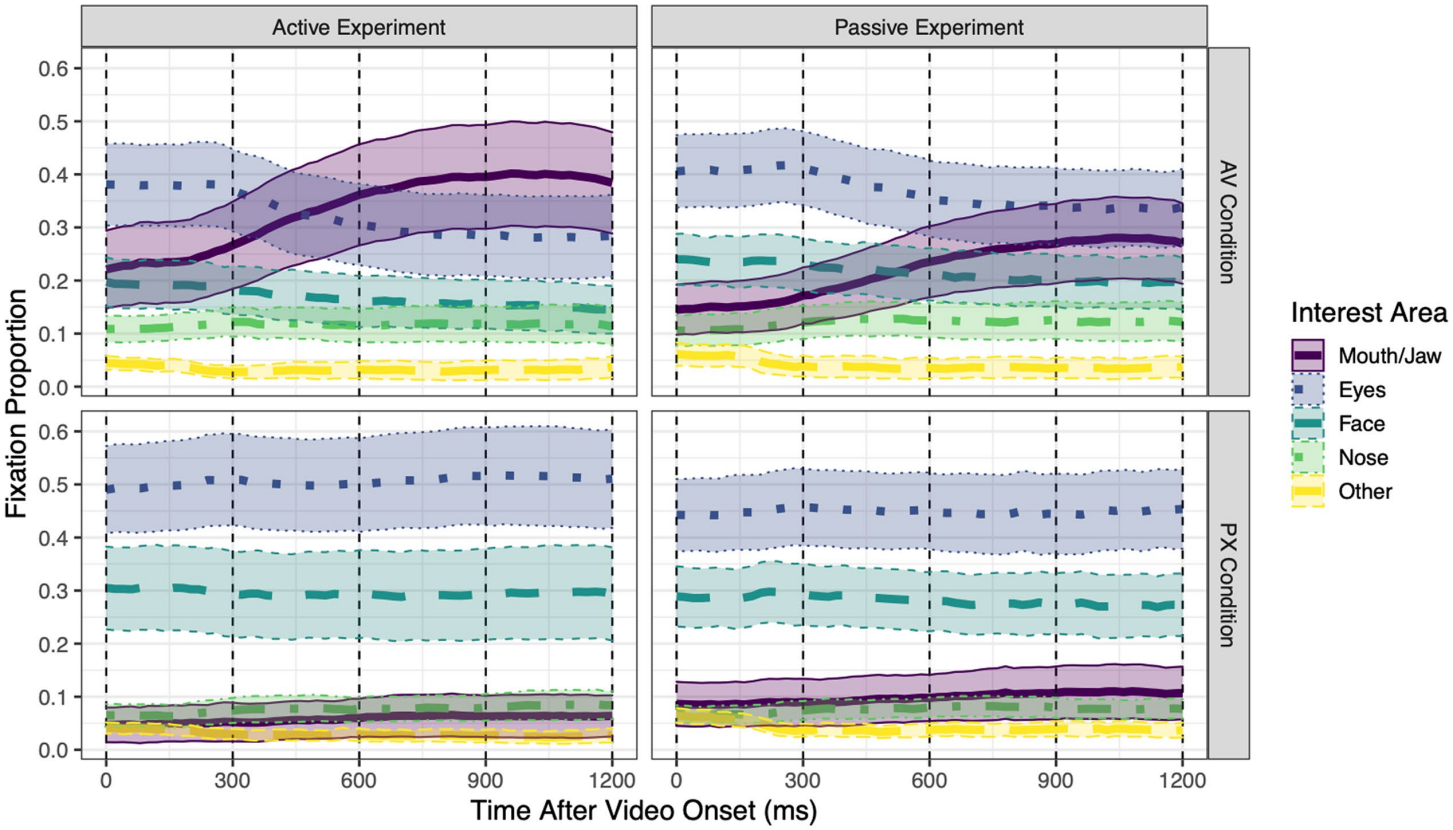
Grand mean proportions of fixations to each of the five interest areas over the course of each trial, separately for each combination of experiment (Active vs. Passive) and condition [audiovisual (AV) or pixelated (PX)]. Within each 20 ms time bin, the sum of fixation proportions to all interest areas is ≤1 (fixations away from the face are not shown). Error ribbons around each line represent 95% confidence intervals. Dashed vertical lines at 300 ms intervals represent the four time windows of interest in the analysis.

Fixations were analyzed over the course of the trial (to provide a dynamic measure of differences in looking patterns to articulators as the speaker produces the /ba/ or /*a*/). A Time Course (Binning) report was used to export the data. This report binned time into 20 ms bins and excluded samples that fell outside of the four predefined interest areas during blinks or saccades. All further analyses were conducted in *R* (R Core Team, 2013).

Prior to analysis, fixations to each interest area were averaged within four consecutive, non-overlapping 300 ms bins. A bin width of 300 ms was used for consistency with prior work (Irwin and Brancazio, 2014) and because the resulting bins appropriately segmented the visual and auditory information, corresponding to the initial rest position (0–300 ms), the mouth opening prior to the consonant closing gesture (300–600 ms), the consonant closing gesture (600–900 ms), and the peak mouth opening for the vowel (900–1,200 ms).

Trial-level analyses were conducted with linear mixed-effects models using the *lme4* package (v. 1.1–21; Bates et al., 2015). One model was fit to each of the two interest areas (Mouth/Jaw and Eyes); the dependent variable for that model was the proportion of fixations to that interest area in each time window on each trial. All models had fixed effects of experiment (two levels: passive = −0.5, active = +0.5), condition (two levels: pixelated (PX) = −0.5, audiovisual (AV) = +0.5), time window (a continuous, centered variable, with contrast weights −1.5, −0.5, +0.5 and + 1.5 so that adjacent time windows were separated by 1), stimulus (two levels: standard /ba/ = −0.3, deviant /*a*/ = +0.7), and within-task trial number (centered and scaled to have a range of 1); all interactions between these fixed effects; and the maximal random effects structure supported by the data.^3^ To identify the maximal random effects structure, we followed a three-step procedure (for this model and all other supplementary models). First, we used the *bobyqa* optimizer to fit a model with a maximal random effects structure: random intercepts for participants, all within-factor random slopes and their interactions, and correlations between random slopes. If the model did not converge, we removed correlations between random slopes. If the resulting model still did not converge, we identified random slopes accounting for <0.01% of the variance of their associated random factors, then removed all such slopes simultaneously (Bates et al., 2018). For both models, this process retained 23/31 random slopes (as well as the random intercept) and resulted in convergence. Degrees of freedom were estimated using the Satterthwaite method in the *lmerTest* package (v. 3.1–3; Kuznetsova et al., 2017).

Main effects and interactions were evaluated using the *contestMD* function from the *lmerTest* package, which conducted ANOVA-like significance tests while retaining the contrast weights used when fitting the model. For significant effects, follow-up contrasts were applied to the fitted model using the *emmeans* package (v. 1.7.1–1; Lenth, 2021). To account for the fact that one model was fit for each interest area, a Bonferroni correction for two comparisons (adjusted alpha = 0.025) was used for all tests. When pairwise comparisons were conducted between means, the Tukey method was used to control familywise error rate.

Analyses of accuracy data (whether participants correctly identified the sound as /ba/ or /*a*/) were conducted using a similar approach. Response accuracy in the active experiment was analyzed using a generalized mixed-effects model with accuracy as the dependent variable (1 = correct, 0 = incorrect). The model included fixed effects of condition, or mouth visibility (two levels: pixelated (PX) = −0.5, audiovisual (AV) = +0.5), stimulus (two levels: standard /ba/ = −0.3, deviant /*a*/ = +0.7), and within-task trial number (centered and scaled); all interactions between these fixed effects; and the maximal random effects structure supported by the data, as identified using the procedure described above. (The model did not include effects of time window, as accuracy was assessed at the level of the entire trial, or effects of experiment, as there were by definition no button presses in the passive task.) In addition, to characterize performance on a standardized scale, we also computed d′ scores for each participant and mouth visibility condition. (Where performance in a condition with n trials was equal to 0% or 100%, count data were adjusted by 0.5/n to permit d′ calculations.) All participants in eye-tracking analyses were included in accuracy analyses except for one participant whose data contained no button presses due to an equipment malfunction; a small number of other trials with missing responses (0.5%) were also excluded.

As described above, trial number and its interactions with other factors were included in the models to account for changes over the course of each task. However, as we were not directly interested in these effects beyond controlling for them statistically, we do not report any results of trial number effects for eye-tracking analyses. In addition, a main effect of stimulus (standard /ba/ vs. deviant /*a*/) and its interactions were included in analyses of eye-tracking data; however, as only a single effect involving this term reached statistical significance in either model (the other 31/32 such effects had *p* > 0.130), we do not report other (non-significant) effects of stimulus.

## 3. Results

### 3.1. Eye-tracking analyses

Mean proportions of fixations to each interest area over the course of the trial are shown in Figure 3 separately for each experiment and condition. All statistical tests are reported in Table 1; means for compared conditions appear below.

**Table 1:** Statistical tests of eye-tracking should be hyphenated.

Condition	Effect	Experiment	Mouth fixations					Eye fixations				
			*β*	97.5% CI	*df*	*t*	*p*	*β*	97.5% CI	*df*	*t*	*p*
AV	Across time windows	Active - Passive	**10.8%**	**[3.8, 17.8%]**	**75**	**3.44**	**<0.001**	−4.6%	[−13.2, 3.9%]	76	−1.21	0.228
	Increase over time windows	Across active and passive	**4.7%**	**[3.6, 5.7%]**	**75**	**9.80**	**<0.001**	**−3.1%**	**[−3.9, −2.2%]**	**89**	**−8.17**	**<0.001**
		Active - Passive	**1.1%**	**[−0.009, 2.2%]**	**90**	**2.22**	**0.029**	−0.7%	[−1.7, 0.3%]	121	−1.31	0.191
PX	Across time windows	Active - Passive	−4.1%	[−11.1, 2.9%]	75	−1.30	0.198	5.9%	[−2.7, 14.4%]	76	1.54	0.128
	Increase over time windows	Across active and passive	0.7%	[−0.3, 1.8%]	75	1.53	0.131	0.2%	[−0.7, 1.0%]	90	0.45	0.651
		Active - Passive	−0.2%	[−1.4, 0.9%]	90	−0.49	0.624	0.5%	[−0.5, 1.5%]	122	0.96	0.337
AV - PX	Across time windows	Across active and passive	**19.7%**	**[14.2, 25.1%]**	**38**	**8.09**	**<0.001**	**−12.6%**	**[−19.1, −6.1%]**	**39**	**−4.35**	**<0.001**
		Active - Passive	**7.4%**	**[2.2, 12.7%]**	**38**	**3.18**	**0.003**	−5.2%	[−11.7, 1.2%]	39	−1.83	0.075
	Increase over time windows	Across active and passive	**3.9%**	**[2.6, 5.3%]**	**39**	**6.62**	**<0.001**	**−3.2%**	**[−4.3, −2.2%]**	**51**	**−7.01**	**<0.001**
		Active - Passive	*1.6%*	*[−0.1, 2.8%]*	*46*	*2.04*	*0.047*	−1.2%	[−2.9, 0.5%]	59	−1.53	0.132

Stimulus	Effect	Condition	Mouth fixations				
			*β*	97.5% CI	*df*	*t*	*p*
Standard - Deviant	Increase over time windows	AV-PX	**0.8%**	**[0.1, 1.5%]**	**122,185**	**2.68**	**0.007**
		AV	**0.5%**	**[0.07, 1.0%]**	**122,187**	**2.55**	**0.011**
		PX	−0.3%	[−0.7, 0.2%]	122,183	−1.24	0.213

Tests are listed in the row order in which they are presented, except for the significant effects of Stimulus which are presented at the bottom. Tests with significant results after adjusting for two comparisons (*p* < 0.025) are shown in bold; tests with marginally significant results (0.025 < *p* < 0.05) are shown in italics.

To address research questions regarding the pattern of gaze to a speaking face and how this pattern changes depending on whether the speech is task-relevant to the listener, we examine gaze patterns when the mouth was visible (i.e., in the AV condition), as this is typical of most everyday speech.

*Mouth/Jaw interest area.* In the AV condition, fixations to the mouth/jaw area (henceforth referred to as “mouth fixations”) were significantly higher in the active experiment than in the passive experiment (active: 33.0%; passive: 22.2%). Over the course of the trial, mouth fixations significantly increased by 4.7% every 300 ms. This increase was marginally greater in the active experiment than in the passive experiment (active: 5.2%; passive: 4.1%).

*Eye interest area*. In the AV condition, fixations to the eye area (henceforth referred to as “eye fixations”) did not significantly differ across experiments (active: 32.6%; passive: 37.3%). Over the course of the trial, eye fixations significantly decreased by 3.1% every 300 ms. This decrease did not significantly differ between the active and passive experiments (active: −3.4%; passive: −2.7%).

To address research questions, regarding the pattern of gaze to a speaking face when the speaker’s mouth is obscured and how this pattern changes depending on whether the speech is task-relevant to the listener, we examine gaze patterns when the mouth was not visible (i.e., in the PX condition) and compare them to gaze patterns when the mouth was visible (AV condition).

*Mouth/Jaw interest area.* In the PX condition, mouth fixations did not significantly differ across experiments (active: 5.9%; passive: 10.0%). Over the course of the trial, mouth fixations increased by 0.7% every 300 ms, a non-significant effect. The size of this change did not significantly differ across experiments (active: 0.6%; passive: 0.8%).

Comparing conditions, mouth fixations were significantly higher in the AV condition than in the PX condition, an effect that was significantly larger for the active experiment (active: 27.1%; passive: 12.2%). Pairwise comparisons between means revealed that mouth fixations were significantly higher in the active AV condition than in all other conditions, and significantly higher in the passive AV condition than in any PX conditions. The extent to which mouth fixations linearly changed over the course of the trial was greater in the AV condition than in the PX condition. The size of this increase was marginally greater in the active experiment (active: 4.6%; passive: 3.3%), and it was significantly greater for standard stimuli than for deviant stimuli (standard: 4.5%; deviant: 3.7%). Describing this three-way interaction in a different way: In the AV condition, looks to the mouth increased over the course of the trial significantly more for standard stimuli than for deviant stimuli (standard: 5.0%; deviant: 4.5%), whereas in the PX condition, there was no difference between stimuli (standard: 0.5%; deviant: 0.8%).

*Eye interest area*. In the PX condition, eye fixations did not significantly differ across experiments (active: 50.5%; passive: 44.6%). Eye fixations did not significantly change over the course of the trial. This (lack of) change did not significantly differ across experiments (active: 0.4%; passive: −0.08%).

Comparing conditions, eye fixations were significantly lower in the AV condition than in the PX condition, an effect that did not significantly differ between experiments (active: −17.9%; passive: −7.4%). Pairwise comparisons between means revealed that eye fixations were significantly lower in the active AV condition than in any PX conditions, and significantly lower in the passive AV condition than in the active PX condition. The extent to which eye fixations linearly changed over the course of the trial was greater in the AV condition than in the PX condition; the size of this decrease did not significantly differ across experiments (active: −3.8%; passive: −2.6%). When participants were not gazing at the mouth or the eyes, they did look at other parts of the face (cheeks, forehead, and ears) as well as the nose.

### 3.2. Accuracy analyses

**Figure 4:**
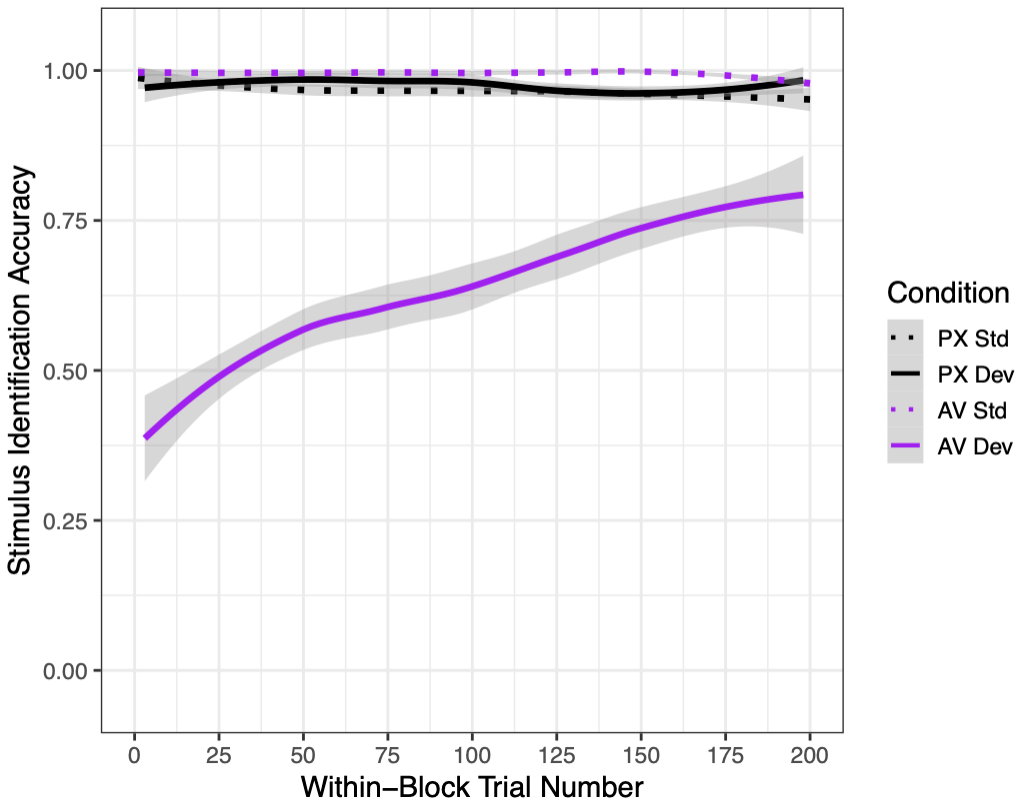
Mean stimulus identification accuracy rates in the active experiment as a function of condition [audiovisual (AV) or pixelated (PX)], stimulus [standard (std) or deviant (dev)], and within-block trial number (1–200). Best-fit lines are LOESS-smoothed; error ribbons represent 95% confidence intervals.

Mean accuracy rates for each condition and stimulus type are shown in Figure 4. In the PX condition, when the mouth was not visible and thus should not have influenced phoneme perception, participants correctly identified 96.6% of standard /ba/ tokens and 97.4% of deviant /*a*/ tokens. In the AV condition, when the mouth was visible and thus could have caused participants to restore /*a*/ to /ba/, participants correctly identified 99.5% of /ba/ tokens but only 64.1% of /*a*/ tokens. These accuracy rates led to mean d′ scores of 4.40 in the PX condition (SD = 0.98) and 3.12 in the AV condition (SD = 1.62). In particular, in the PX condition, all participants except for one had d′ scores >2.00.

Statistical analyses supported these differences, as reported in Table 2. Condition and stimulus interacted to affect accuracy. Pairwise differences between conditions revealed that deviant stimuli in the AV condition were responded to significantly less accurately than stimuli in all other conditions. In addition, standard stimuli were recognized significantly better in the AV condition than in the PX condition, indicating that mouth visibility improved identification accuracy when the visual and auditory signals were consistent.

**Table 2:** Statistical tests of response accuracy in the Active condition.

Effect or contrast	*β*	95% CI	*z*	*p*
Condition × Stimulus	**−7.52**	**[−10.25, −4.78]**	**−5.39**	**<0.001**
AV Dev – (AV Std, PX Std, PX Dev)	**[−6.50, −4.46]**		**[−6.26, −5.01]**	**<0.001**
AV Std – PX Std	**2.03**	**[0.66, 3.40]**	**2.90**	**0.019**
Condition × Stimulus × Trial Number	**6.20**	**[1.91, 10.50]**	**2.83**	**0.005**
Effect of trial number for AV Dev	**7.53**	**[4.99, 10.06]**	**5.82**	**<0.001**
Effect of trial number for (AV Std, PX Std, PX Dev)	[−1.76, 0.03]		[−1.87, 0.02]	>0.061

Tests are listed in the row order in which they are presented. Rows 1 and 4 represent model effects; other rows represent contrasts. In rows in which groups of contrasts are jointly reported, ranges are provided to describe the *β*, *z*, and *p* values for all contrasts in the group. AV, audiovisual; PX, pixelated; Std, standard stimulus; Dev, deviant stimulus. DV = Accuracy of button press responses in the active experiment. Tests with significant results (*p* < 0.05) are shown in bold.

Lastly, although we did not perform a systematic investigation of adaptation effects, we note that there was a significant three-way interaction between condition, stimulus, and trial number. Specifically, accuracy rates improved over the course of the block for deviant stimuli in the AV condition but did not significantly change for stimuli in any other condition.

## 4. Discussion

Using this phonemic restoration paradigm within passive and active experiments, which included an AV condition with a full speaking face and a PX condition in which articulatory features were unavailable, we asked several research questions. We hypothesized that within the active and passive AV condition, gaze patterns would reveal a high level of fixations to the mouth; however, greater fixations to the mouth would be apparent in the active experiment where participants were required to press a button for the deviant /a/ sound. In the PX condition, it was hypothesized that greater fixations would occur to the eyes, when visible articulatory information from the mouth was unavailable. Button press accuracy data was hypothesized to be higher within the active PX condition, in which articulatory features from the face were not available, hence unable to influence a phonemic restoration effect for deviant /a/ stimuli. This phonemic restoration paradigm allowed for the investigation of gaze patterns when articulatory features are both present and absent and within contexts which required no response from the participant or actively required the participant to discriminate between stimuli.

As expected, across the passive and active experiments, there were significantly more fixations to the mouth in the AV condition compared to the PX condition. This is consistent with previous research suggesting that when articulatory information is available from a speaking face, adults gaze more to the mouth, and ignore other facial features such as the eyes (Lansing and McConkie, 2003; Võ et al., 2012). When comparing gaze patterns within the same condition between the two experiments (i.e., AV passive vs. AV active, PX passive vs. PX active), there were significantly more fixations to the mouth in the active experiment relative to the passive experiment only when the mouth was visible (i.e., in the AV condition). This was expected as the active experiment required the participant to discriminate between the speech tokens by pressing a button for each of the stimuli (/ba/ or /*a*/). The active experiment engendered a listening situation which required the participant to make judgements regarding the stimuli and perhaps aligned more closely with real-life speaking situations in which listeners are required to actively disambiguate unclear or distorted speech. The increased gaze to the mouth in the active condition may indicate that participants actively searched for supporting visual speech information from the mouth to support speech perception.

Two areas of interest, the eyes and the mouth/jaw, were chosen based on previous research (Pelphrey et al., 2002; Nguyen et al., 2009; Võ et al., 2012; Yi L. et al., 2013; Åsberg Johnels et al., 2022). Researchers have previously noted that gaze patterns form a “T” shape, with the most time spent looking at the eyes then the mouth or nose when looking at faces at rest (Yarbus, 1967; Pelphrey et al., 2002). Others have found that when looking at a speaking face, adults fixate on the eyes but move to the mouth once the speaker begins talking (Lansing and McConkie, 2003; Võ et al., 2012). Across the 300 ms time windows, fixations to the mouth/jaw area were highest in the active AV condition. There were more fixations when the mouth was visible and when the environmental demands necessitated the participants to discriminate between speech tokens and provide a button press response. Thus, when there is information that can be gleaned from the mouth/jaw area, and there is a higher demand/requirement to discern speech, adults take advantage of this input.

In terms of gaze patterns in the PX condition, when the mouth was not visible, there was no significant difference between the amount of fixations to the eyes between the passive and active experiments (mirroring the lack of a difference in fixations to the mouth). There were, however, differences between the AV and PX conditions: Participants looked significantly more at the eyes when the mouth was pixelated (PX condition) than when the mouth was visible (AV condition). As eye gaze is considered an automatic orienting cue that can be used rapidly in face-to-face interactions, perhaps when visual articulatory information is not present, participants seek other communicative information in order to maximize whatever information is left to make optimal decisions and potentially resolve a temporary ambiguity (Hanna and Brennan, 2007). Other researchers have found that people focus on the eyes when making judgements about age and fatigue (Nguyen et al., 2009). Looks to the eyes when articulatory information of the mouth is not available is particularly relevant when people wear masks. Although articulatory information may not be available when a speaker wears a mask, listeners may attempt to glean other information such as a speaker’s intent or emotional state during a communicative exchange (Buchan et al., 2007; Schurgin et al., 2014).

In terms of accuracy data (measured by a button press response within the active experiment), participants demonstrated *lower* accuracy levels within the AV condition compared to the PX condition, suggesting that participants did experience a phonemic restoration effect when the presence of articulatory information from the mouth was available. This result is consistent with our previous studies on phonemic restoration in adults and children (Irwin et al., 2017a,b, 2022). Identification of the *standard stimuli* within the active experiment were significantly better in the AV condition compared to the PX condition. This is consistent with the observation that looks to the mouth increased over the course of each AV trial with standard, but not deviant, stimuli. The deviant stimuli within the AV condition were correctly identified *significantly* less than the deviant stimuli in the PX condition. Taken together, these results suggest that the participants were less likely to identify differences between auditory tokens in the presence of the speaking face. This is because visual information from a speaking face influences what a person hears, and therefore, in this experiment, leads to a decreased ability to discriminate the *deviant stimuli*. This finding aligns with previous studies in which suggests when there is a mismatch in audio and visual information from a speaking face listeners precepts may be changed or influenced (McGurk and MacDonald, 1976; Irwin et al., 2017b). These findings suggest that visual information of the mouth improved or enhanced perception when both the auditory and visual stimuli were in sync, or perhaps that the visual stimulus in the AV condition led to an overall response bias toward perceiving standard stimuli. Interestingly, in addition, accuracy results for the deviant stimuli /*a*/ within the AV condition improved over the course of the trials. In other words, given more trials which included a visual speaking face with the attenuated /*a*/ token, the participants were able to discriminate the /*a*/ token better over time, despite the absence of feedback. Therefore, the phonemic restoration effect diminished or lessened with practice. It is possible that when provided with several trials of competing/different sensory stimuli (in this case differing visual and auditory tokens), the practice effect allows for greater differentiation between different stimuli.

The results of the present study are consistent with previous studies which report that when audiovisual information is present within a listening situation, adults will specifically gaze at the mouth area to extract articulatory information to support the processing of speech (Lansing and McConkie, 2003; Võ et al., 2012; Yi A. et al., 2013; Hadley et al., 2019; Banks et al., 2021). This gaze pattern was most significant when environmental demands necessitated that the participants discriminate between auditory tokens. These findings are consistent with previous research on audiovisual integration in contexts where listeners are required to disambiguate speech information, or grossly aligned with studies of gaze patterns in speech in noise or degraded speech in that listeners seek visual cues from the mouth to support speech processing and perhaps understanding (Vatikiotis-Bateson et al., 1998; Buchan et al., 2008; Yi L. et al., 2013; Yi A. et al., 2013; Hadley et al., 2019; Banks et al., 2021). Our accuracy results indicated that adults had better performance in identifying the standard /ba/ stimulus in the AV active condition, which suggests that the speaking face significantly improved the identification of the standard stimuli when the audio and visual articulatory information were in sync. This adds to the literature by suggesting that the presence of visual articulatory information indeed improves or enhances speech perception to some extent in different speaking contexts (Sommers et al., 2005; Smiljanic et al., 2021). These results align with studies demonstrating improved accuracy of speech signals in the presence of visual cues (Banks et al., 2021; Yi et al., 2021; Lalonde et al., 2022).

Further, these findings have important clinical and real-world consequences such that general face-to-face communication in which articulatory information is available has the potential to enhance speech perception. This may be particularly important for persons with hearing loss, language impairment, or those learning a new language. Although our study investigated neurotypical adults and used simple syllables, further work is needed to specify how the presence of a speaking face affects speech perception at the word and sentence level in both children and older adults where prevalence of hearing impairment and or speech/language impairment is increased.

Previous researchers have shown the negative impact of missing visual articulatory information in speech perception. However, researchers have not specifically addressed where listeners look to glean any residual visual information to strengthen the understanding of speech when key areas are obscured. Although these studies have specifically measured speech perception within masked/unmasked situations, to the authors’ knowledge, no studies have directly investigated gaze patterns to speaking faces where the mouth is present or absent, yet other facial features are present. Thus, our study provides additional information about where listeners look when articulatory information is present or absent. This is particularly relevant in light of the COVID-19 pandemic which brought about the use of masks in all areas of society where communication took place. Masks specifically covered mouth and nose regions. Our results suggest that when visual information from the mouth is absent, listeners compensate by gazing more at the eyes.

### 4.1. Future directions

The paradigm used in this study, although related, does not directly apply to daily communication (i.e., conversations) as this set of experiments focused on syllables. Thus we would caution about overgeneralizing these results. Future research is needed to extend this study to understand if there are differences in eye gaze patterns in the presence and absence of articulatory information during more natural communication (i.e., words and sentences). These results also have important implications for both neurotypical and clinical populations. In terms of neurotypical populations, previous studies have suggested that older adults may integrate information across all senses that are available to them. Older adults have been found to perform similarly to younger adults within audiovisual speech discrimination (Cienkowski and Carney, 2002; Tye-Murray et al., 2007; Winneke and Phillips, 2011) while auditory (Dubno et al., 1984; Gordon and Allen, 2009; Füllgrabe et al., 2015) and visual (Feld and Sommers, 2009; Tye-Murray et al., 2016) identification show age-related declines. Other researchers have noted audiovisual integration declines with age (Honnell et al., 1991; Dancer et al., 1994; Sommers et al., 2005; Ross et al., 2007; Tye-Murray et al., 2010). Further research is needed to determine how declines (or lack thereof) in the integration of audiovisual sensory information, impact communication in older adults who may be affected by age-related hearing loss or cognitive decline. Regarding clinical populations, there is a substantial amount of literature on gaze patterns to faces within autism. Our team has previously demonstrated that children with autism are less visually influenced than neurotypical controls in tasks that involve phonetic processing of visual speech (Irwin et al., 2011; Irwin and Brancazio, 2014). Ongoing work will examine the role of eye gaze for persons with autism where articulatory information is either present or absent and when environmental demands increase the need to perceive changes in speech-related information.

### 4.2. General conclusions

Audiovisual integration is a critical aspect of speech processing. Results of the current study suggest that adults gaze specifically at the mouth in listening situations and that gaze to the mouth increases when environmental demands require that a listener perceive differences in speech stimuli. When visual information is unavailable, listeners look specifically to the eyes when perceiving a speaking face. Finally, adults are most likely to experience a phonemic restoration effect when presented with asynchronous audiovisual input, in this specific case an attenuated token of the consonant syllable /ba/. The presence of audiovisual stimuli enhances accuracy of a spoken syllable over time, providing further evidence that the presence of visual articulatory information can enhance speech perception.

## Data availability statement

The raw data supporting the conclusions of this article will be made available by the authors, without undue reservation.

## Ethics statement

The studies involving human subjects were reviewed and approved by Yale University IRB, Southern Connecticut State University IRB, and University of Rhode Island IRB. The participants provided their written informed consent prior to participation in the study. Written informed consent was obtained from the individual(s) for the publication of any identifiable images or data included in this article.

## Author contributions

JI and NL contributed to the conception and design of the study. LC programmed and troubleshot the experiment. AB, VH, DK, LC, NL, and JI collected participant data. AB and JM processed and cleaned the data. DK performed the statistical analyses. AB, DK, VH, JI and JM wrote the manuscript. All authors contributed to the article and approved the submitted version.

## Funding

The study was supported by NIDCD grant R15DC013864 awarded to JI (PI).

## Conflict of interest

The authors declare that the research was conducted in the absence of any commercial or financial relationships that could be construed as a potential conflict of interest.

## Publisher’s note

All claims expressed in this article are solely those of the authors and do not necessarily represent those of their affiliated organizations, or those of the publisher, the editors and the reviewers. Any product that may be evaluated in this article, or claim that may be made by its manufacturer, is not guaranteed or endorsed by the publisher.

## Footnotes

^1^Restoration occurs when the replacement of a phoneme results in an illusory perception of the missing speech sound. Our paradigm includes elements of a McGurk effect given that the phoneme is not completely absent, but weakened, therefore providing mismatched audio and visual input. However, the paradigm itself has been show to elicit the restoration of a weakened consonant, essentially aligned more with a restoration effect rather than a new percept.

^2^ https://osf.io/ehvg8/

^3^The contrast weights for /ba/ and /*a*/ stimuli reflect the fact that /ba/ was presented on 70% of trials: each trial is weighted equally in analyses, rather than each stimulus type. Due to the scales used for the continuous variables, effects of time window correspond to the change in fixations between consecutive 300 ms time windows, and effects of trial number correspond to the change in fixations between the first and last trials in each block.
